# Quantitative exploration of factors influencing psychotic disorder ailments in Nigeria

**DOI:** 10.1016/j.dib.2017.07.046

**Published:** 2017-07-24

**Authors:** Adebowale O. Adejumo, Nehemiah A. Ikoba, Esivue A. Suleiman, Hilary I. Okagbue, Pelumi E. Oguntunde, Oluwole A. Odetunmibi, Obalowu Job

**Affiliations:** a Department of Mathematics, Covenant University, Ota, Nigeria; b Department of Statistics, University of Ilorin, Ilorin, Nigeria

**Keywords:** Psychotic disorder, Bipolar, Vascular dementia, Minimal brain dysfunction, insomnia, schizophrenia, X-squared statistic, Binary logistic regression

## Abstract

In this data article, records on demographic data, family problem issues, as well as results of medical tests from five major classes of psychotic disorder namely: bipolar; vascular dementia, minimal brain dysfunction; insomnia; and schizophrenia, were collected on 500 psychotic patients carefully selected from the pool of medical records of Yaba Psychiatric Hospital, Lagos, Nigeria, for the period of 5 years, between January 2010 and December 2014, were examined. X-squared Statistic was used to examine each of psychotic disorders to identify demographic (age, gender, religion, marital status, and occupation) and family issues (loss of parent, history of such ailment in the family (family status), divorce, head injury, and heredity of such ailment (genetic) factors that influence them. A clear description on each of these psychotic disorders (bipolar; vascular dementia, minimal brain dysfunction (MBD), insomnia and Schizophrenia) was considered separately using tables and bar diagrams. Data analysis results are as follows: firstly, 40.2%, of the 500 psychotic patients tested positive to bipolar, 40.6% to insomnia, 75.0% to schizophrenia, 43.6% to MBD and 69.2% to vascular dementia. Secondly, female patients were more prone to all the psychotic indicators than their male counterpart except in MBD. Thirdly, the oldest age group (> 60 years) is more prone to bipolar and insomnia ailments, while the mid age group (30 – 60 years) is prone to schizophrenia and vascular dementia, and the youngest group (< 30 years) is prone to MBD. Lastly, the factors that influence the ailments are listed: **bipolar** (age, occupation, marital status, divorce, and spiritual consultation); **insomnia** (age, occupation, marital status, divorce, and spiritual consultation); **schizophrenia** (age, occupation, religion, marital status, hereditary, and divorce); **MBD** (gender, age, occupation, and marital status); and **vascular dementia** (history of the ailment and spiritual consultation). Bipolar and insomnia are influenced by the same set of factors, which implies that any patient having one is most likely to be at risk of having the other.

**Specification Table**

**Table:** 

Subject area	Medicine
More specific subject area	Psychotic Disorder, Psychiatry, Neuroticism, Psychosis
Type of data	Tables and figures
How data was acquired	Unprocessed secondary data
Data format	Processed as patient by patient records on Demographic variables, Family problems issues and Test results from fiveclasses of Psychotic Disorder indicators
Experimental factors	Data obtained from Yaba Psychiatric Hospital, Yaba, Lagos
Experimental features	Computational Analysis: Contingency Tables, X^2^ statistic for test of independence, Histogram, Bar diagram
Data source location	Yaba Psychiatric Hospital, Yaba, Lagos State, Nigeria
Data accessibility	All the data are in this data article as a supplementary data file
Software	SPSS Statistical program and Microsoft Excel

**Value of the Data**

• The data on psychotic disorder patients could be useful for the government to monitor the mental health activities of the population, most especially the youth.
• The data will be useful in survival analysis and demographic studies.
• The data can be useful for educational purposes and health assessment studies.
• The data is useful in the study of epidemiology of psychiatry and public health.
• Several known models, for example, binary logistic regression, multinomial logistic regression, multiple regression and probability fit can be applied which provide alternatives to analysis with X^2^ statistic.
• The data analysis results may fuel further investigations on the area for example the gender and age differences in the manifestation of the various ailments.
• Comparative analysis may be carried out using the data and other previous studies on psychotic disorder ailments.
• The prevalence and distribution of the psychotic disorder obtained from the data analysis can help in psychiatric counselling and management of psychotic episodes.
• The quality of the data could be improved by increasing the number of variables or modifying the inherent variables.

## 1 Data

The data for this paper were obtained from Yaba psychiatry hospital, Yaba, Lagos state, Nigeria, being the medical records of 500 psychotic patients for a period of five years between January 2010 and December 2014. The data are 16 variables classified as demographic variables (gender, age, marital status, occupation and religion); family problems/ issues (history of ailment in the family, loss of parents, family hereditary of the ailment, head injury, spiritual consultation, and divorce); and medical test result for five psychotic disorder indicators (bipolar, vascular dementia, minimal brain dysfunction, insomnia, and schizophrenia). The data can be accessed as Supplementary data.

The descriptions of the ailments are given below.

**Definition**

• Bipolar disorder: This is a form of brain disorder that causes unusual and uneven shifts in mood, energy and activity levels and the ability to effectively perform routine tasks. This can manifest as manic, hypomanic and depressive (mood) episodes.
• Insomnia: This is a problem of the brain being able to effectively coordinate sleep patterns resulting to long duration of sleeplessness and loss of sleep drive.
• Schizophrenia: This is a form of severe mental disorder that affects the thinking, feelings and behavioural ability of the person. The symptoms include: hallucinations, thought and movement disorders, delusions, reduced speaking, inconsistency in routine activities, reduced reasoning capability, and inattentiveness and so on.
• Minimal brain dysfunction (MBD): This is a neurodevelopmental disorder which is characterized by under control of emotions, activity and behavior and cognitive difficulties in learning and writing.
• Vascular dementia: This ailment bears close resemblance in symptoms with Alzheimer's disease. It is a gradual decline in cognitive capability due to obstructions to blood flow to the brain. Symptoms include: speech and vision impairment, confusion, anxiety and disorientation.

### 1.1 Age distribution of the psychotic patients

Statistical summary of the age distribution of psychotic patients is presented in Table 1.

**Table 1:** Summary statistics of the age distribution of the psychotic disorder patients.

Statistic	Value
Mean	37.16
Standard error of mean	0.689
Median	34.00
Mode	34.00
Standard deviation	15.401
Skewness	0.495
Standard error of skewness	0.109
Kurtosis	-0.455
Standard error of kurtosis	0.218
Minimum	6
Maximum	86
Lower quartile	24.00
Upper quartile	46.75

It can be seen that the average age of these patients is 37 years. The youngest and the oldest psychotic patients are 6 and 86 years old respectively.

**Fig. 1:**
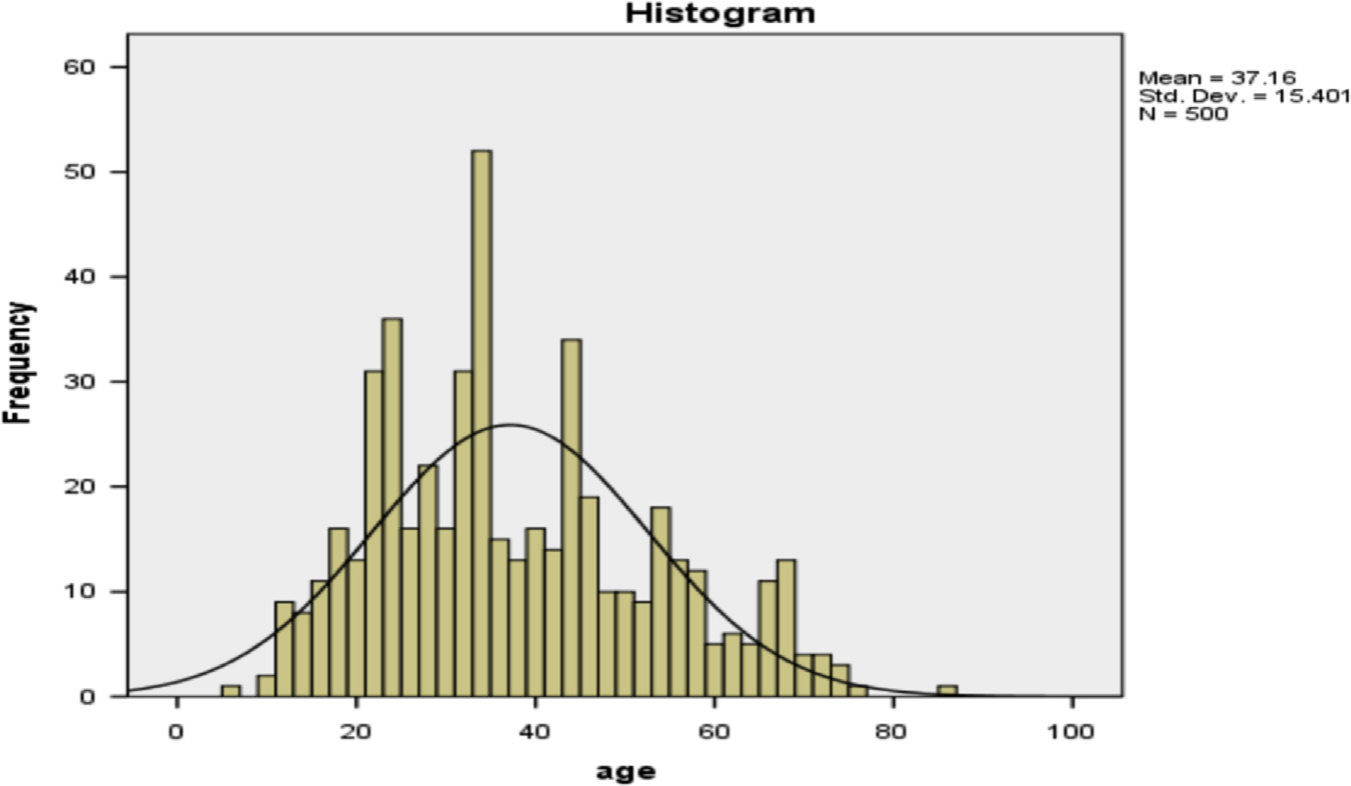
Histogram of age distribution of psychotic disorder patients in Yaba psychiatry hospital between 2010 and 2014.

Histogram for age distribution is presented in Fig. 1.

It can be observed from Fig. 1 that the age distribution is almost normally distributed with mode and median the same (34 years), only mean (37 years) is slightly different. With the aid of Fig. 1, the age of the patients is classified into three different categories, namely: less than 30 years, between 30 and 60 years inclusive, and greater than 60 years.

### 1.2 The demographic variables of the psychotic patients

The detailed demographic variables of the psychotic patients investigated for the five disorders are summarized in Table 2.

**Table 2:** Summary of demographic variables of the patients in relation to the five psychotic ailments the patients tested positive.

Demographic Variable		Bipolar	Insomnia	Schizophrenia	MBD	Vascular dementia
Gender	Male	85	87	203	140	161
	Female	116	116	222	78	185
Age group	< 30	42	44	173	104	125
	30 – 60	76	76	204	102	154
	>60	83	83	48	12	67
Religion	Christianity	82	84	193	98	148
	Islam	93	93	177	91	157
	Others	26	26	55	29	41
Occupation	Artisan	53	56	131	57	100
	Civil Servant	44	43	62	22	49
	Force	6	6	21	14	14
	Retired	38	38	7	4	26
	Student	29	29	113	68	80
	Unemployed	31	31	91	53	77
Marital status	Married	148	148	220	94	190
	Single	53	55	205	124	156
Overall		201 (40.2%)	203 (40.6%)	425 (75.0%)	218 (43.6%)	346 (69.2%)

From Table 2, it can be seen that 40.2%, of the 500 psychotic patients tested positive to bipolar, 40.6% to insomnia, 75.0% to schizophrenia, 43.6% to MBD and 69.2% to vascular dementia. This implies that schizophrenia is the most incident ailment among the psychotic patients, followed by vascular dementia and others.

It can also be seen that female patients were more prone to all the indicators than their male counterpart except in MBD. However, on average 135 males out of a total of 233 male psychotic patients, which is 57.9% and 143 females out of a total of 267 females are tested positive which is 53.6%.

Bipolar disorder and insomnia are most prevalent in the oldest age group (> 60 years). Schizophrenia and vascular dementia are most prevalent in the mid age group (30–60 years) and MBD is most prevalent in the mid age group in the youngest group (< 30 years). In general, mental disorder is most prevalent in the mid age group and least in oldest age group.

No general statement is made as the sample size is small and inadequate to infer the overall psychotic population. The data is obtained from the patients and is not intended to attribute any disease incident or prevalence to any religion. Yaba Psychiatry hospital is one of several psychiatric hospitals in Nigeria.

All the five psychotic disorders are most prevalent in the artisan group and least in the force group.

The pressure of marriage may be a reason why psychotic ailments are prevalent among the married patients and externalizing behavior which is more prevalent among the youths may be the reason why MBD is most prevalent among the single patients.

### 1.3 The family issues distribution of positive test results

The psychotic patients that tested positive to the five psychotic disorder ailments are classified according to their family issues/problems and are shown in Table 3. Each of the family problem issue's variables are cross classified with number of those that were tested positive for the five psychotic disorder indicators.

**Table 3:** Summary of the five psychotic disorder ailments tested positive by family issues and presence of head injury.

Variable		Bipolar	Insomnia	Schizophrenia	MBD	Vascular dementia
History in family	No	91	92	203	102	150
	Yes	110	111	222	116	196
Heredity	No	115	115	245	126	195
	Yes	86	88	180	92	151
Loss of parent(s)	No	85	85	175	95	140
	Yes	116	118	250	123	206
Divorce	No	164	166	384	194	309
	Yes	37	37	41	24	37
Head Injury	No	162	164	341	174	282
	Yes	39	39	84	44	64
Spiritual consult	No	49	50	134	67	45
	Yes	152	153	291	151	301
Overall		201	203	425	218	346

### 1.4 Proportion of true positives

In addition, the proportions of those that are really positive for each of the family problem issues are presented in Table 4. That is, those that had the history of the ailment in their family, inherited the ailment, lost their parents, divorced, had head injury or consulted spiritualist and at the same time tested positive on any of the five psychotic disorder ailments.

**Table 4:** Summary of proportion of psychotic patients that have ‘YES’ option on any of the family problem issues and tested positive to the five psychotic disorder ailments.

Proportion of true positives	Bipolar	Insomnia	Schizophrenia	MBD	Vascular dementia
History	0.547	0.547	0.522	0.532	0.566
Hereditary	0.428	0.433	0.424	0.422	0.436
Loss of parent(s)	0.577	0.581	0.588	0.564	0.595
Divorce	0.184	0.182	0.086	0.110	0.107
Head injury	0.194	0.192	0.198	0.202	0.185
Spiritual consult	0.756	0.754	0.685	0.693	0.870

On the average, 54.3% were having history of the ailment in their family and tested positive for any of the five psychotic indicators, 42.9% inherited the ailment from their family and tested positive for any of the five psychotic indicators, 58.1% lost their parent(s) and tested positive for any of the five psychotic indicators, 13.4% were divorced and tested positive for any of the five psychotic indicators, 19.4% had head injury and tested positive for any of the five psychotic indicators, and 75.2% consulted spiritualist and tested positive for any of the five psychotic indicators.

## 2 Methods and materials

Several studies have been conducted on the psychotic disorder ailments. [1], [2], [3], [4], [5], [6], [7], [8], [9], [10], [11], [12], [13], [14], [15], [16], [17], [18], [19], [20], [21], [22], [23], [24], [25], [26], [27], [28]. Similar data articles on medicine that applied statistical tools can be helpful, readers are refer to [29], [30], [31], [32], [33], [34], [35], [36], [37], [38], [39], [40].

Contingency table is a rectangular table having *I* rows for categories of *X* and *J* columns for categories of *Y.* The cells of the table represent the *IJ* possible outcomes. In order to test for independent or association between the two categories X and Y, we used X-squared statistic which is approximately Chi-squared distribution.

Table 5 presents contingency table for just five (5) different combinations out of fifty-five (55), of any of the five psychotic indicators and any one of the demographic variables (5 of them) or family problem issues (6 of them) stated in this paper.

**Table 5:** Contingency data summary.

Demographic variable		Bipolar		Insomnia		Schizophrenia		MBD		Vascular dementia	
		**N**	**P**	**N**	**P**	**N**	**P**	**N**	**P**	**N**	**P**
Gender	Female			151	116						
	Male			146	87						
Age	< 30					8	173				
	30 – 60					10	204				
	>60					57	48				
Religion	Christianity	140	82								
	Islam	126	93								
	Others	33	26								
Occupation	Artisan							87	57		
	Civil servant							51	22		
	Force							7	14		
	Retired							42	4		
	Student							52	68		
	Unemployed							43	53		
Marital status	Married									91	190
	Single									63	156
Overall		299	201	297	203	75	425	282	218	154	346

N = Negative, P = Positive

Table 6**,** also presents the estimates of the X^2^ statistic for each of the combinations in contingency Table 5.

**Table 6:** Summary of the X^2^ estimates of five psychotic disorder indicators against demographic factors (with p-value in bracket).

Demographic variables	Bipolar	Insomnia	Schizophrenia	MBD	Vascular dementia
Gender	2.511 (0.113)	1.924 (0.165)	1.545 (0.214)	48.225 **(<0.0001)**⁎	0.002 (0.963)
Age group	89.619 **(<0.0001)**⁎	86.573 **(<0.0001)**⁎	160.892 **(<0.0001)**⁎	59.768 **(<0.0001)**⁎	2.199 (0.333)
Occupation	63.852 **(<0.0001)**⁎	61.422 **(<0.0001)**⁎	198.550 **(<0.0001)**⁎	47.252 **(<0.0001)**⁎	9.504 (0.091)
Religion	1.818 (0.403)	1.313 (0.519)	6.779 **(0.034)**⁎	1.140 (0.566)	1.308 (0.520)
Marital status	41.493 **(<0.0001)**⁎	38.749 **(<0.0001)**⁎	22.643 **(<0.0001)**⁎	26.868 **(<0.0001)**⁎	0.756 (0.385)

⁎ Significant at 5% level of significance.

**Fig. 2:**
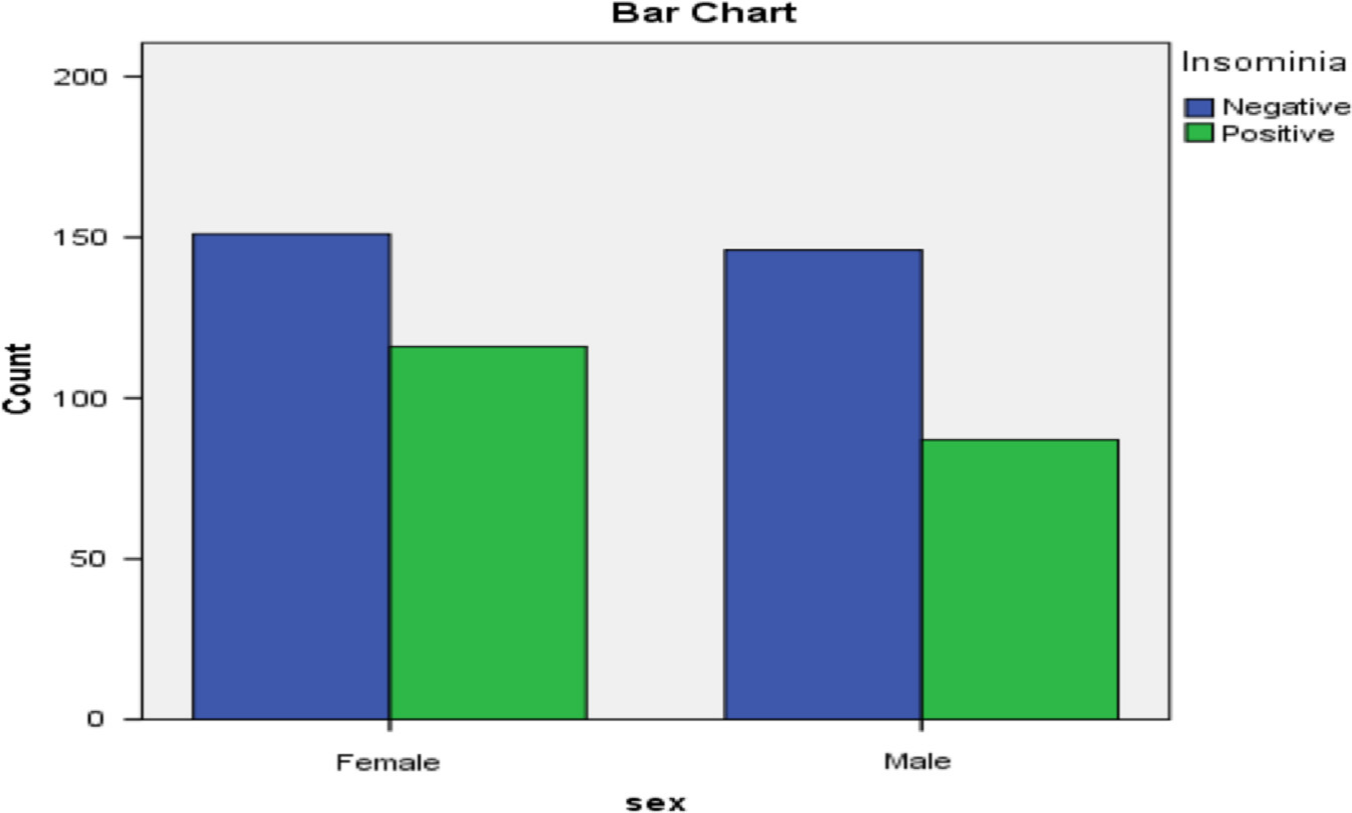
Bar diagram for gender and Insomnia.

**Fig. 3:**
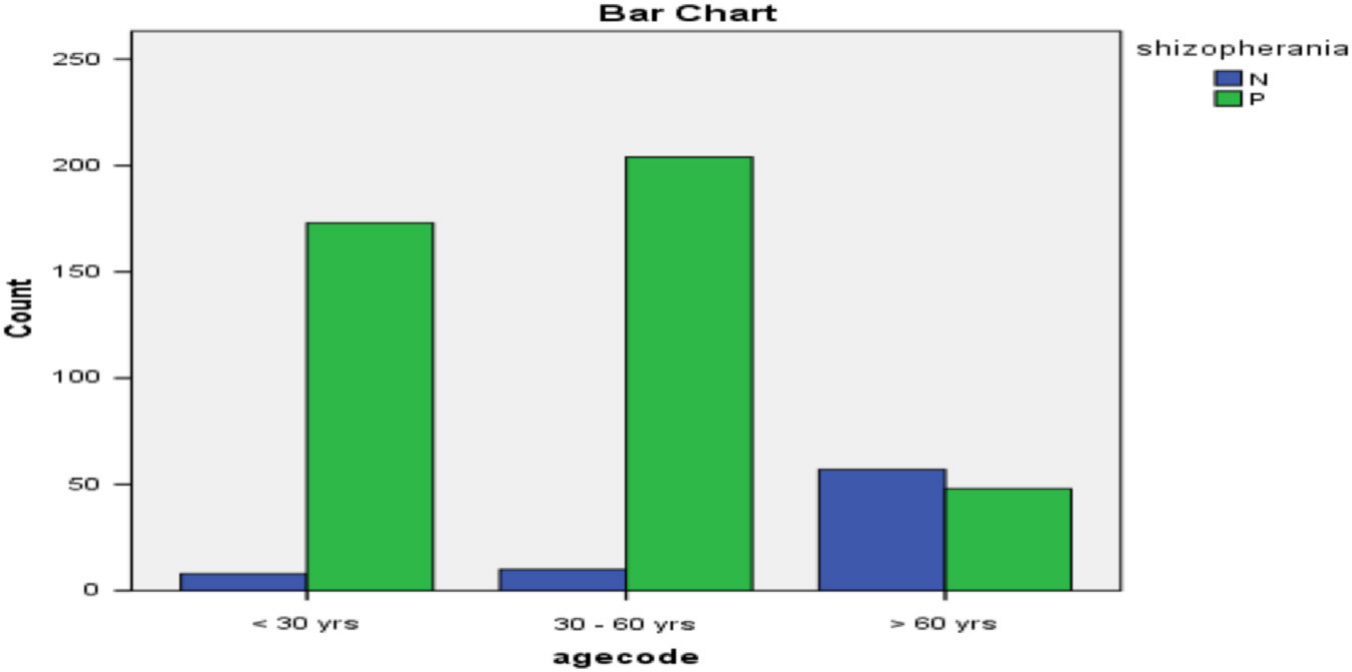
Bar diagram for age-group and schizophrenia.

**Fig. 4:**
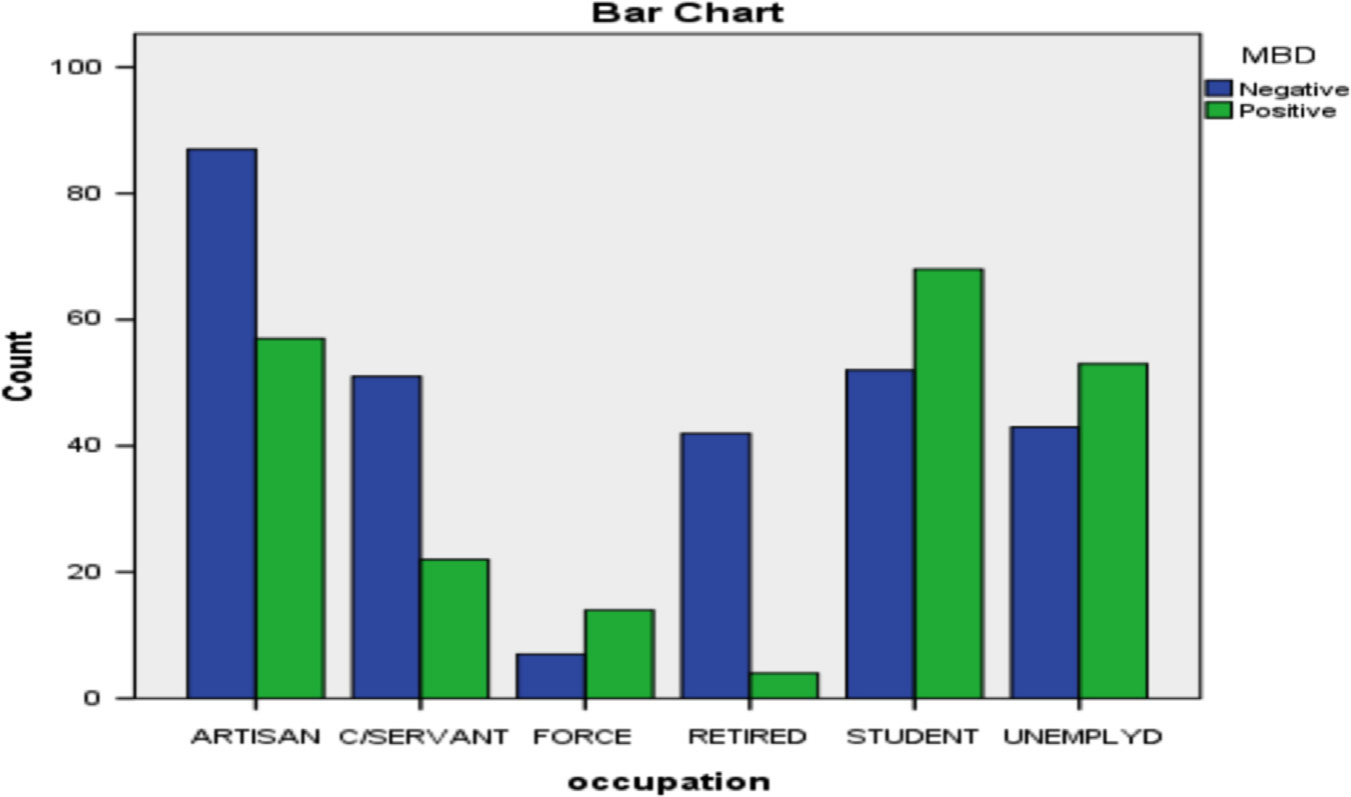
Bar diagram for occupation and MBD.

**Fig. 5:**
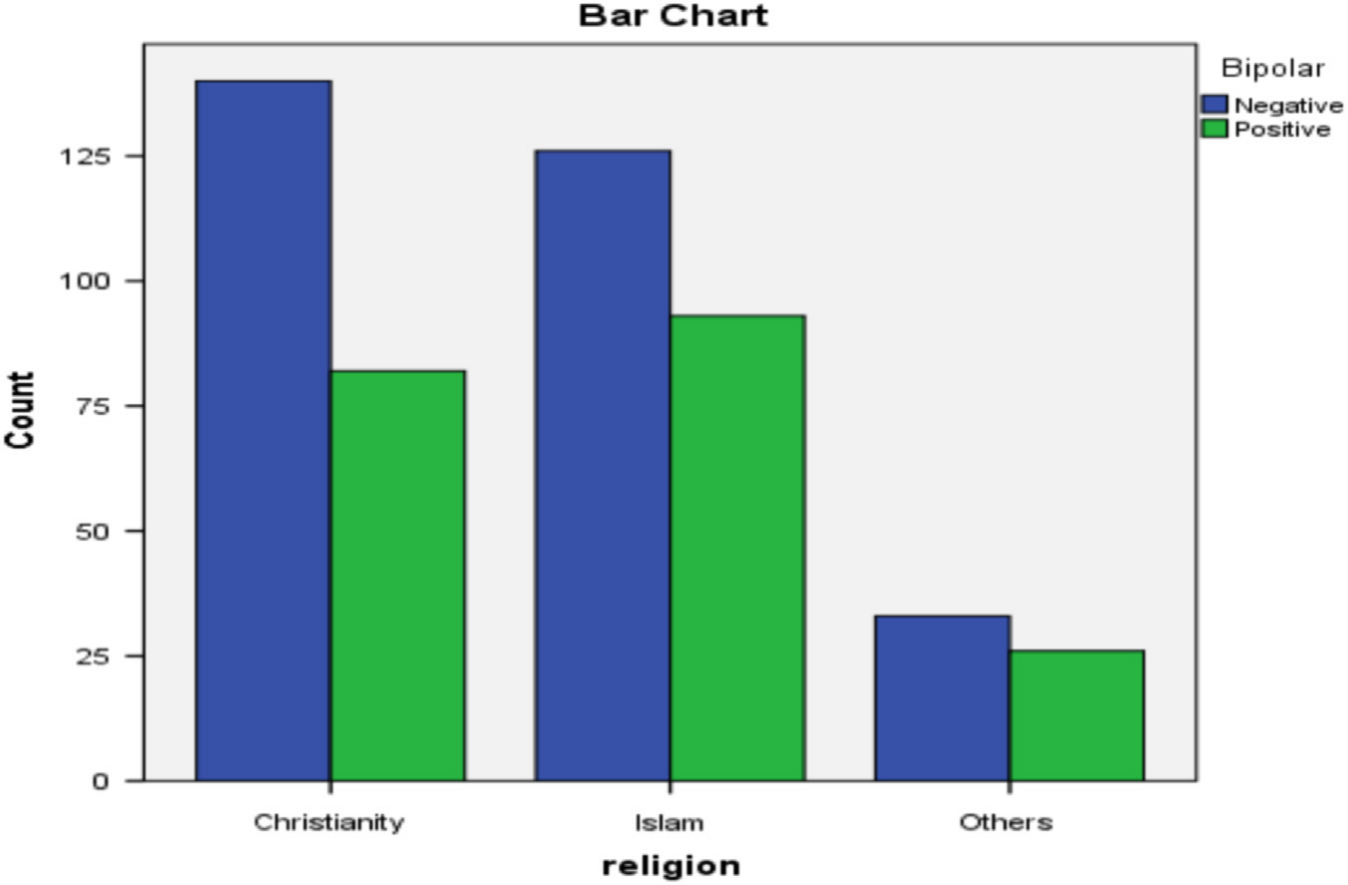
Bar diagram for religion and bipolar.

**Fig. 6:**
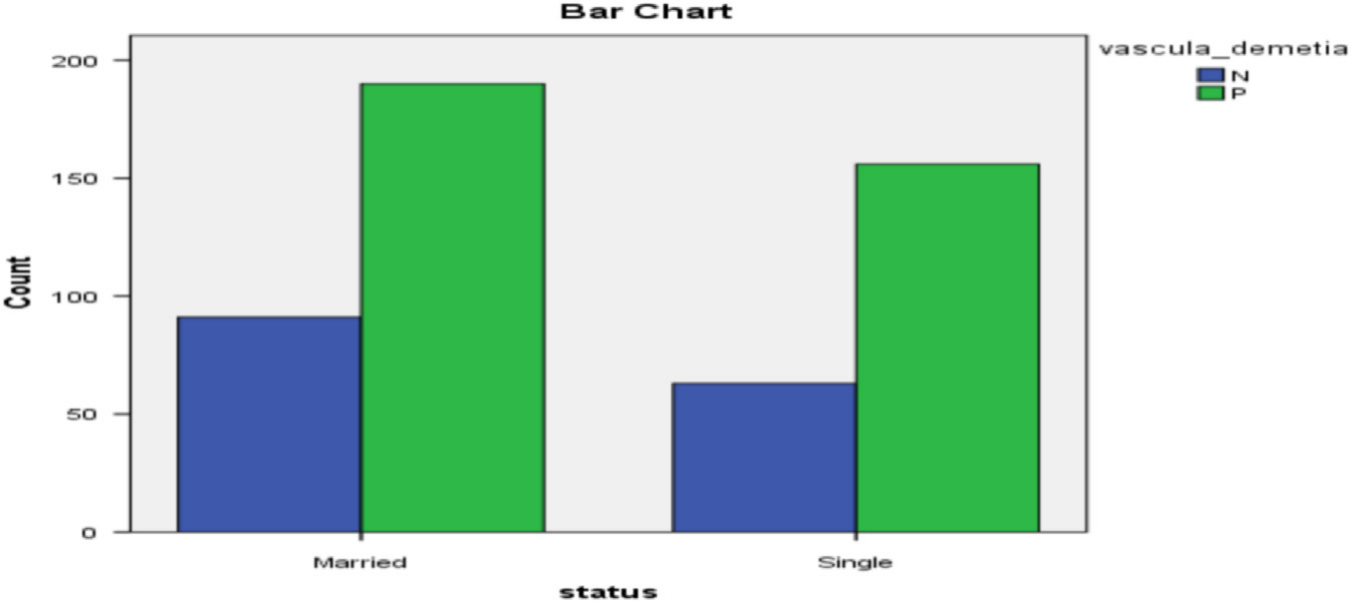
Bar diagram for marital status and vascular dementia.

Fig. 2, Fig. 3, Fig. 4, Fig. 5, Fig. 6 present the bar diagram for each of the combinations in contingency Table 5.

Table 6, Table 7 present the summary X^2^ statistic estimates for the five psychotic indicators, and the five demographic variables and six family problem issue indicators respectively with their p-values.

**Table 7:** Summary of the X^2^ estimates of five psychotic disorder indicators against family problem /Issues (with p-value in bracket).

Family Problem/issues	Bipolar	Insomnia	Schizophrenia	MBD	Vascular dementia
History of ailment in the family	0.116 (0.733)	0.106 (0.744)	2.791 (0.095)	0.054 (0.816)	3.664 **(0.050)**⁎
Hereditary	0.272 (0.602)	0.100 (0.752)	3.919 **(0.048)**⁎	0.626 (0.429)	0.142 (0.706)
Loss of parent(s)	0.498 (0.480)	0.308 (0.579)	0.709 (0.400)	1.621 (0.603)	0.002 (0.966)
Divorce	13.070 **(<0.0001)**⁎	12.547 **(<0.0001)**⁎	14.854 **(<0.0001)**⁎	0.359 (0.549)	1.815 (0.178)
Head injury	0.080 (0.777)	0.380 (0.546)	1.727 (0.189)	0.485 (0.486)	0.680 (0.795)
Spiritual consult	6.127 **(0.013)**⁎	5.735 **(0.017)**⁎	1.152 (0.283)	0.003 (0.954)	163.751 **(<0.0001)**⁎

From the data analysis shown in Table 6, Table 7**,** it can be seen that the factors that influences:

**Bipolar**: age, occupation, marital status, divorce, and spiritual consultation.

**Insomnia**: age, occupation, marital status, divorce, and spiritual consultation.

**Schizophrenia**: age, occupation, religion, marital status, hereditary, and divorce.

**MBD:** gender, age, occupation, and marital status.

**Vascular dementia**: history of the ailment and spiritual consultation.

Bipolar and insomnia are influenced by the same set of factors, which implies that any patient having one is most likely to have the other.

⁎ Significant at 5% level of significance.

## Footnotes

Transparency document Transparency data associated with this article can be found in the online version at http://dx.doi.org/10.1016/j.dib.2017.07.046.
