# Supplementary material for: Quantitative exploration of factors influencing psychotic disorder ailments in Nigeria
Source: Data Brief. 2017 Jul 24;14:175–85. doi: 10.1016/j.dib.2017.07.046 (PMC5537424; doi:10.1016/j.dib.2017.07.046)
Supplement: Supplementary file 1 — Supplementary material [file mmc1.pdf]

# COVENANT UNIVERSITY
## COLLEGE OF SCIENCE AND TECHNOLOGY
## DEPARTMENT OF MATHEMATICS

CANAANLAND, KM 10, IDIROKO ROAD
P.M.B 1023, OTA, OGUN STATE, NIGERIA
www.covenantuniversity.edu.ng, mat.covenantuniversity.edu.ng

**EXTERNAL MEMO**

**To:** Editor, Data in Brief
**From:** Corresponding Author
**Date:** 14th July, 2017
**Subject:** **Conflict of Interest**

I thereby declare the absence of any conflict of interest among the authors.

The authors have read the final draft and unanimously agreed that the paper be sent for review.

**Hilary I. Okagbue**
Department of Mathematics, Covenant University, Ota.
Google Scholar: Hilary Izuchukwu Okagbue
Research Gate: Hilary Okagbue
Live DNA: 234.14453
Scopus ID: 56438006100
ORCID: 0000-0002-3779-9763
